
JOURNAL INFORMATION
==============================
NLM Title Abbreviation: Inter Econ
Journal ID: Inter Econ
NLM Journal ID: 101086399

Inter Economics

ISSN: 0020-5346

ARTICLE INFORMATION
==============================
PMCID: PMC7584855
DOI: 10.1007/s10272-020-0917-x
Article ID: 917
Article version: 1
Subjects: Forum

The Eurozone as a Trap and a Hostage: Obstacles and Prospects of the Debate on European Fiscal Rules

Costantini Orsola orsola.co@gmail.com

Macroeconomic and Development Policies, United Nations Conference on Trade and Development (UNCTAD), Palais des Nations, 8–14, Av. de la Paix, 1211 Geneva 10, Switzerland
Electronic publication date: 2020 Oct 24
Print publication date: 2020
Volume: 55
Issue: 5
First page: 284
Last page: 291
Copyright: © The Author(s) 2020
License: Open Access: This article is distributed under the terms of the Creative Commons Attribution 4.0 International License (https://creativecommons.org/licenses/by/4.0/).
Open Access funding provided by ZBW — Leibniz Information Centre for Economics.
License URL: https://creativecommons.org/licenses/by/4.0/

issue-copyright-statement: © The Author(s) 2020

==============================

The debate about what, whether and when fiscal rules should be reinstated is of existential relevance for the European Union for at least two main reasons. First, the recovery is, or should be, a public good that governments must take care of (Shapiro, 2019). Previous attempts to privatise the burden of recovery have failed and a quick return to austerity entails serious economic, social, environmental, and probably political risks. Second, the European fiscal framework impacts much more than just the size of the budget deficit. It influences defining aspects of the social contract, including universal access to healthcare, pensions, inheritance taxes and social mobility, youth employment and the gender divide.

As much as economists, as citizens, should contribute to the discussion, the problem is essentially political (Roncaglia, 2015; Di Majo, 2020). But no fair intra-European debate can occur if the financial stability of the euro area is not somewhat secured. This, alas, may amount to a conundrum.

Circumstantial actions have so far allowed the eurozone bumblebee to keep flying. But key factors of fragility are entrenched in its architecture. If the risks associated with a monetary union without fiscal transfers have been highlighted for a long time,[1] some additional factors were introduced during and after the debt crisis of 2010. In fact, what was a lost decade for growth, was a productive decade in terms of legislative innovation both nationally and at the EU level (Costantini, 2017; Clency, 2019).

Some innovations came from a functional, but far from uncontroversial, expansion of the pre-existing rules. Those were, if belated, often vital to the survival of the euro and include the ECB public bonds purchasing programmes. In contrast, the more political evolution seemed to push in an opposite destabilising direction, as were the private sector involvement, and the establishment of an incomplete banking union. The fiscal constraints were tightened and forced upon some member countries as a condition for accessing the rescue programmes. All of these reforms took advantage of and then reinforced a pecking order among the member countries, ultimately establishing austerity as a common social and political project (Truger, 2013; Storm and Nastepaad, 2016). Nationally, most countries underwent regressive reforms of public spending – especially of pensions, healthcare and other social policies – and the labour market.

While destabilising, this restriction of national policy autonomy has not established institutions capable of providing prompt response to crises. With the exception of the ECB, only intergovernmental action can do that, which helps shape the debate as a clash of national interests. Even if the current crisis has opened up possibilities previously unimaginable, the negotiations at the July heads of state meeting proved to be difficult, revealing how maintenance or acquisition of national privileges are prioritised over common solutions. Meanwhile, the European Parliament was essentially silenced (Bordignon, 2020).

Crucially, the agreed measures, including the temporary suspension of some ECB and SGP constraining rules and the provision of additional funds for recovery, do not mitigate the risk associated with the EMU architecture that still exposes its members and their banking systems to potentially devastating liquidity and solvency risks (Ferguson and Kane, 2020). Proof is the continuous capital flight out of Italy recorded on the Target 2 system, which has intensified since the beginning of the pandemic (Minenna et al., 2018). In this situation, in which avoiding financial panic depends on a shaky agreement between current governments (Tooze, 2020) and on the ECB keeping its current course, any legitimate concern or debate that risks throwing the bumblebee off balance – such as on the national uses of common funds, or on monetary policy – is instantly 'weaponised', i.e. strategically abused on one side to generate market reactions, and branded as irresponsible on the other.

At once a trap and a hostage, the eurozone and the EU are stuck: the obstacles to a more stable union are political, but no fair political debate can occur without a financial stabilsation because the risk of instability cripples the political debate.

A first step, thus, is to consolidate some basic functional innovations to guarantee 'minimal sovereignty conditions' for countries to be able to represent their electorates at the EU tables, and for the political debate within countries to happen unconstrained – even when this makes them unpopular with big financial investors and credit agencies or other EU members. We cannot have a repetition of what induced Mario Monti in 2012 to agree to an incomplete banking union. The minimal conditions are addressed in this article.

The second step could amount to a new constitutional moment for the EU and lead to a reform of economic governance. The process requires free the fiscal policy framework from its technocratic chokehold and reconnect it to its social and political function. Obscurely estimated targets and benchmarks must be abandoned and the mystification of independent fiscal authorities rejected. As much as it would be nice to separate the technical from the political in economic policy, as auspicated by the European Fiscal Board (2019), it is simply impossible and usually a strategy to favour interests that do not command democratic support. A better option, as a recent proposal by the Macroeconomic Policy Institute (IMK, 2020) of the Hans-Böckler-Stiftung suggests, is to set up tables of discussions between experts, political representatives and the social partners. The second and final section of this article discusses some recent proposals for a reform of the Stability and Growth Pact and possible alternatives. In particular, I argue that it is necessary to drop the idea of a trade-off between sustainability of public finances and macroeconomic stabilisation, and that any rule must take into account the asymmetric problems posed by membership in a monetary union without fiscal transfers. I re-state the merits of a budget structure based on large built-in automatic stabilisers, which can be agreed up on in advance and then left alone to do their job over the cycle, accompanied by a separate capital budget for long-term investment plans with a full employment and other social and environmental targets that can, *ex post*, create the savings that guarantee sustainability.

1 Godley (1992), Goodhart (1998), Parguez (1999), and Simonazzi and Vianello (1999).

The challenge is existential: In a world ravaged by a pandemic, an export-led growth strategy is simply not feasible (United Nations Conference on Trade and Development [UNCTAD], 2020). As the euro appreciates, the eurozone members, especially Germany, France and Italy, must rise to the challenge and contribute to global development as engines of growth rather than subtract resources, savings and people from the rest of the world.

### Minimal sovereignty conditions: The ECB as a lender of last resort and the reform of the banking union

The European debt crisis revealed – once again – how central bank policies are crucial to protect the national fiscal space from self-fulfilling prophecies of debt unsustainability. Speculative appetite can nullify the most 'responsible' action on the part of governments, whatever that is believed to be. This, of course, raises questions about the political role of monetary policy institutions and on the meaning of their 'independence'.

The direct trigger of the European debt crisis was the disproportionate rise in the public debt burden of some smaller peripheral economies of the eurozone, due to the failure of member countries to share responsibility for stabilising the national banking sectors in 2008 and 2009. But it was the ECB architecture and policy orientation, that prevented it from being the lender of last resort and induced Trichet to prematurely raise interest rates, which actively produced instability, creating the opportunity for a speculative attack that pushed the yields to unsustainable heights. Even at that point, the central bank was unable and unwilling to act to normalise market pricing, by purchasing the bonds herself. It was then that financial contagion also hit countries, such as Italy, whose debt had not changed in a significant way, as a proportion to GDP, during the crisis.

In fact, the post-2010 European 'rescue' programmes were based on the idea that austerity would reassure the markets, but not only did they fail to reduce the spreads, they also crippled the economy. By the time Draghi pronounced his famous "whatever it takes", the peculiar European double-dip recession was already well underway and bound to leave permanent scars on the welfare and demographics of the hardest hit regions.

For instance, Portugal asked for a bail out on 11 April 2011. The 'Troika' required commitment to a programme to cut deficits by more than six percentage points in three years, which included extensive privatisations, a forced reduction of the public and private employee compensation as well as of pensions, VAT and income tax increases, cuts to all social services and transfers, and a shift of social contribution security burden away from selected companies (especially in the export sector) to workers (Weeks, 2019).

Figure 1
**Employment in Portugal, 1993-2019**

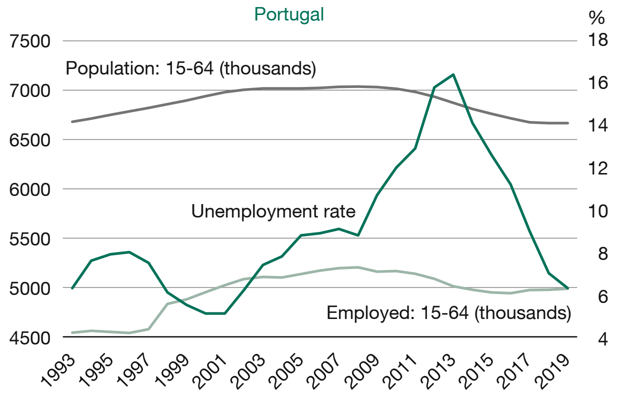

Source: AMECO.

During that time, the rating agencies repeatedly downgraded the country's bonds, eventually to junk, generating further tensions and effectively closing Portugal's access to market finance. Its growth rate was negative for two consecutive years. The unemployment rate reached 16.4% in 2013, just as a mass emigration started which, together with the increase in discouraged workers, contributed to contain unemployment figures (Figure 1). Portugal still (before the burst of the pandemic) payed a larger share of output in interest than it did in 2010.

Unfortunately, Draghi's expression of commitment and the subsequent quantitative easing (QE) programmes did not always prevent market tensions, arising from intra-EU political disputes, from suddenly affecting yields, nor were they sufficient to close the spreads. Part of the reason is the capital key rules and the fact that the ECB always seems to act discretionally and at the limit of legitimacy. For instance, recently, the newly appointed president Christine Lagarde's 'slip' reminded the world that the ECB could change its stance at any time.

This discretion is not denied and actually positively viewed by many at the ECB such as ECB board member Isabel Schnabel (2020), who recently reassured us that "sovereign bond markets are still performing their disciplinary role, in spite of the ECB's asset purchases." In fact,

> Although interest rates have fallen broadly across advanced economies in recent years, risk premia in euro area sovereign bond markets have not disappeared. Today, for example, 10-year yield spreads on Italian government bonds over their German equivalents are high-

> er than when the ECB started to purchase government bonds in early 2015. They also remain responsive to idiosyncratic news. The marked response of Italian sovereign bond yields to the 2018 episode of political instability, which by the way was not countered by monetary policy, underlines the disciplinary role played by financial markets. (Schnabel, 2020)

Of course, we are left in the dark about why Italy, which has primarily run budget surpluses since 1995, except in 2009, and has not once defaulted on debt payments in its entire history, does not qualify as a credible borrower as much as Germany, Portugal or the Netherlands (Cesaratto and Zezza, 2018; Storm, 2019). We are also left wondering how the concept of political instability is defined, if not as 'whatever upsets financial markets'.

But what Schnabel said is nothing new; it is written down in the Treaties. The statute of the ECB and its restrictive mandate were designed precisely to regulate member states with respect to their fiscal policy; that is, to allow the financial markets to orient fiscal choices by determining the *space* within which they can move. This idea presumes that the markets are effective at pricing correctly and ultimately resolve risk differentials by directing capital flows; this also ignores or downplays the possibility of speculation. After the debt crisis, the ECB has not been able to ignore that possibility altogether but, as Schnabel's speech indicates, it still considers it an exception.

On the contrary, price movements have represented a risk in themselves, largely independent of real conditions: speculative thinking lies at the heart of how finance works (Lincei, 2020). After all, it was speculation that allowed large imbalances between core and periphery to accumulate during the 2000s, which ultimately became unsustainable, causing a major bank crisis in Spain, Portugal, Ireland and Germany – but not in Italy (O'Connell, 2015).

Indeed, the entire eurozone system is based on the idea that freedom-limiting moral hazard comes from the state, not the market (Buchanan and Wagner, 1977). In practice, it is geared towards letting speculation strive. A perfect case in point is the banking union which, in 2012, was presented as a three-pillar project that would allow for the recovery and full integration of the European banking system. It included a common supervision system, a procedure for rescue or resolution of banks in crisis, and a common insurance on deposits. In reality, "the banking union generated huge asymmetries and unfair competitive conditions across the Eurozone" (Giacchè, 2017), furthering fragmentation.

First, the failure to this day to institute the third pillar, i.e. the European insurance on deposits, implied that member countries remained responsible for protecting savers, hence maintaining the risk of a doom loop between banking and a sovereign debt crisis. Furthermore, the resolution process requires that a fixed portion of the uninsured assets be used to absorb the losses (bail in). The decision was taken only after many European countries had extended unprecedented financial support to their national banks, thus significantly distorting competition. With holders of subordinated bonds suddenly facing higher risks, the measure triggered a bank run, intensifying otherwise manageable difficulties that banks were facing. The rigidity of the bail-in rule is rather unique in the international landscape and its financial convenience has been questioned even by the International Monetary Fund (2018).

If the second pillar created risks where there were none, the first pillar made sure that the pre-existing high systemic risk from some of the larger banks in the area would grow undetected. In fact, not only does the common banking supervision system cover various national banking systems very unevenly, it also crucially downplays and underestimates market risk (linked to financial activity), putting a much greater emphasis on credit risk (Kane, 2017; Biondi and Del Barrio, 2019).

In the words of the Chair of the European Central Bank's Supervisory Board himself, Andrea Enria (2019):

> The banking union has been successful in promoting a more resilient banking sector. But it is still failing to deliver an integrated domestic market for banking business. Rather than smoothing idiosyncratic shocks to individual Member States, the banking sector still operates as a shock amplifier.

Hence, if a broad reconsideration of the principles behind the structure of the banking union and supervisory mechanisms is needed, at a minimum, the third pillar should be instituted to guarantee the "minimal sovereignty conditions" necessary to even begin such discussion. Given the obvious system risks that the absence of the third pillar poses, its institution should be considered a mere functional innovation.

That might be controversial, but the challenge is not new to European institutions. In fact, the acceptable limits of 'functional' innovation in monetary policy have also been repeatedly questioned by the German Constitutional Court, which claims that the ECB's decisions regarding the QE were taken beyond the powers granted. In the most recent decision, which re-states the prerogative of member states to conduct a judicial review of the ECB's policies, the Court demanded that the ECB provide further information to assess if the second QE programme violates the democratic principle by which fiscal policy is a national responsibility (Righi, 2020).

The German Court's ruling is appropriate in so far as it poses the question of monetary and fiscal sovereignty in political and constitutional terms. Unfortunately, it puts the cart before the horse. As conceived from the outset in the eurozone, monetary policy is not neutral with respect to fiscal policy, but aims at influencing it with action that is independent only in accordance with a specific economic theory, i.e. independent from elected bodies and reliance on the markets' less-than-perfect risk assessment. No government is sovereign in absence of a lender of last resort that follows an explicit mandate to protect the fiscal and political space and, hence, no fair intra-European discussion can occur without such an explicit commitment from the ECB.

### Reform of the Stability and Growth Pact

In March of this year, the Stability and Growth Pact was temporarily suspended to allow for extraordinary budget deficits during the COVID-19 crisis. However, the spectre of a reinstatement looms over the EU, affecting the choices that governments make today.

The economic rationale for the institution of the Stability and Growth Pact in 1997 was well explained by Isabel Schnabel (2020), in the same previously mentioned speech:

> The Stability and Growth Pact was designed to ensure that governments would pursue sound fiscal policies and that public debt would remain low and stable, or at least converge to such levels in a gradual and credible manner. The fiscal framework of the European Union was meant to shield the ECB from fiscal dominance and protect its independence.

What Schnabel did not say is that the SGP failed its purpose.

With the ECB forced to resort to unconventional policies, the European fiscal framework induced pro-cyclical budgetary measures that reduced growth, increasing unemployment and reducing tax receipts, and intensified inequality, with negative effects on the capacity to reduce the debt-to-GDP ratio and on the capacity of the ECB to reach the inflation target. In the past decade, countries in the Southern periphery experienced a dramatic impoverishment (Celi et al., 2015). Germany – if compared, for instance, with the US – had a disappointing economic performance and, in 2019, was already on the brink of a contraction.

European fiscal rules are flawed in many respects; the estimates that they are based on need constant revision and deny, by assumption, that lower debt-to-GDP ratios can result from fiscal expansion (see IMK, 2020, for a review of the flaws). They are founded on the assumption that actual output, with its cyclical fluctuations, naturally gravitates around a normal or potential level that is supply-determined, that is, it does not respond to changes in demand. However, in practice, this unobservable potential output is calculated as more or less sophisticated average, thus itself moving with the cycle and absorbing fluctuations in actual output which are then attributed to changes in the supply-side factors (Palumbo, 2013; Heimberger and Kapeller, 2017). This explains why in the EU, discussions on cyclically adjusted budgets have reached, as we know, grotesque dimensions, with unemployment rates above 20% considered normal. Already in April, the European Commission has revised downwards the estimates of potential income for member countries, therby reducing the fiscal space that it considers to be non-inflationary in 2021 (Heimberger, 2020).

In addition to their poor technical performance and their macroeconomic failure, the severe fiscal supervisory procedures have intensified instability 'mechanically' with abrupt admonishments of the national governments, often in the form of recurring letters that create financial tensions and political pressure (Costantini, 2018).

An even more fundamental problem exists: in a monetary union, with countries with different levels of inflation, productivity and industrial specialisation, symmetric budget constraints can hardly be appropriate (Irvin and Izurieta, 2011; Horn and Watt, 2017).

But the other reason for the adoption of the Stability and Growth Pact was the mere fact that the much sounder alternative, i.e. the creation of a fiscal union, was not politically acceptable. In addition, the presence of an 'external constraint' proved useful for governments implementing unpopular austerity reforms. Even today, the legitimate concerns about the use each country makes of the common recovery funds could be solved by giving more power to the Parliament and putting it in charge, in a federal setting, of a bigger budget, but national pressures prevent that outcome. It is unclear whether the creation of a fiscal union can be postponed for much longer. But, for now, the Stability and Growth Pact is still seen as a necessary compromise.

In the face of widespread criticisms about its formulation, however, the Commission initiated a review process and assigned the European Fiscal Board the task of producing a report. The 2019 report (European Fiscal Board, 2019) starts with the premise that a trade-off exists between debt sustainability and macroeconomic stabilisation and envisages a simplified set of rules and a recommendation to set up an independent fiscal authority. The recommended rules include a 'fiscal anchor' corresponding to a debt-to-GDP ratio and a declining path towards it, and an 'indicator of fiscal performance', that is a ceiling on the growth rate of net (non-cyclical) primary expenditures for countries with debt in excess of 60% of GDP.

> Concretely, the growth rate of the expenditure ceiling would be capped by the trend rate of potential output growth … Member States with a debt ratio below 60% of GDP would not be subject to a net expenditure ceiling, but would still have to observe the 3% deficit. (European Fiscal Board, 2019, 88)

Naturally, the impact of this set of rules would depend on various details, such as how potential output is estimated and the timeframe for the debt ratio reduction. The report itself notes some caveats:

> To bring about a convergence to the 60% of GDP debt norm in the Treaty over a relatively short time span of, say, 15 or 20 years (as envisaged in the current rule) … Italy and Portugal would have to run primary budget surpluses … in the order of 4-5% of GDP over a decade or more. (European Fiscal Board, 2019, 89)

Ultimately, struggling to find a solution to the asymmetric burden that debt reduction requires of different member countries, the Board envisages shifting to a procedure of country specific assessments and recommendations, with the involvement of technical institutions.

Unfortunately, the report's suggestions do not seem to distance themselves fundamentally from the current rules. For instance, the idea that countries with debt-to-GDP ratios higher than 60% should spend less in order to reduce it remains foundational to the plan.

Several objections can be raised: for instance, that the 60% threshold is arbitrary (Pasinetti, 1998). Moreover, there is no macroeconomic basis for arguing that a ratio should reduce rather than simply stabilise, in a country issuing debt in its own currency, with a lender of last resort (Blanchard, 2019). But even if there were such a need, an expansionary fiscal policy is the fastest way to achieve that goal (Ciccone, 2013; UNCTAD, 2020). And actually the countries that have the highest debt ratio at the moment are, with the exception of France, those that implemented the harshest austerity in the past ten years, which caused a deterioration of the quality of the public services offered and of the productive capacity: they are the countries that should be spending the most.

The report, moreover, remains mute about the lack of tax harmonisation in the EU that makes it difficult for countries to increase tax rates on some categories of incomes and wealth, and induces privilege to spending cuts, with regressive consequences.

As a form of flexibility, the suggested new framework would allow for a series of escape clauses that guarantee the unconstrained ability to spend during an emergency, such as the one we are living. This is consistent with the neo-Keynesian recipe for timely, targeted and temporary fiscal expansion (Bernanke, 2008). However, it is precisely underspending in normal times that produces more violent cycles and intensifies the need for discretionary spending which often cannot quickly compensate for a lack of investment over time. A perfect case in point is the current crisis, which exposed underfunding of the health care facilities (Prante et al., 2020).

In that regard, European fiscal rules have led to a consequence that would seem paradoxical. Designed originally to minimise discretion, they actually induced a reduction of the built-in counter-cyclical budget elements, such as tax progressivity, social transfers and buffers, precisely so that the budget deficit would tend to increase less in response to downward income fluctuations. Similarly, permanent programmes, such as health care and education, which are countercyclical in the downward part of the cycle, were a favoured target of austerity. Hence, a reform of the Stability and Growth Pact should take its initial vocation seriously and de-emphasise reliance on discretionary spending in favour of stronger automatic stabilisers and farsighted plans that will smooth the cycles and reduce the need for extraordinary deficits.

Of course, the economy does not normally oscillate around its optimal state, and crises can have long-term negative effects on growth. Hence, automatic stabilisers alone cannot guarantee that the path to full employment is maintained (Costantini, 2015). For this reason, the idea of a golden rule for public investment (Truger, 2016; IMK, 2020), which would exempt it from the restrictive deficit targets, should be seriously considered. In an entirely new fiscal framework, however, countries could be asked to institute separate capital investment budgets[2] with a full employment and other targets.[3] In other words, the capital budget should be allowed to run a deficit until they achieve full employment, as well as a carbon footprint reduction target or any other preferred social or environmental objective. In the meantime, a process of EU-wide harmonisation should help re-orient European policies from self-defeating competition over labour costs or tax rates into a mutually benefitting coordination. In this framework, there would be no need for an expenditure rule, as the current budget would balance over the cycle.

2 See for instance Myrdal (1939), Kregel (1985), Seccareccia (1995).
3 Others suggested the reform and involvement of the European Investment Bank (Arestis et al., 2001; Watt, 2016).

## Conclusions

In this paper, I argue that the European fiscal framework needs to be reformed to guarantee that the EU spends its way to recovery, re-emerges from the previous prolonged stagnation and fully contributes to the relaunch of global growth on a sustainable path. Abandoning the pro-austerity bias that is hard wired into the current Stability and Growth Pact and its arsenal of estimates is also the only way to stabilise public finances, allowing for expansionary budgets that boost growth and create, *ex post*, enough savings, or *fiscal space*, to guarantee sustainability.

I discuss how the most influential proposals still accept the existence of a trade-off between financial sustainability and macroeconomic expansion, and limit growth-friendly budget flexibility to the creation of escape clauses for occasional discretionary spending. On the contrary, I argue that a new pact should reinforce and harmonise automatic stabilisers and create national capital investment budgets with a full employment and a set of social and environmental targets. A successful strategy of public investments should allow the current budget to balance over the cycle, taking care of the long-term stimulus to expand the economy's potential.

Budget policies, however, need to be de-technicised and democratised (Di Majo, 2020). In fact, both expansionary and restrictive fiscal policies have distributive effects and can deeply transform the economy as well as society: they are fundamentally political. Indeed, the largest part of the stimulus in 2008 and 2009 was a direct or indirect subsidy to the banking and financial sector or to the immediate damages it had done. On the contrary, the post-2010 programmes targeted key social institutions and public services, such as the labour market and the health care sector. Today, as the debt ratios spike, talks of further privatisations and pension reforms have already started.

Unfortunately, however, the entire EU system suffers from a democratic deficit and is geared towards maintaining austerity. In fact, the persistence of elements of financial fragility that (now) operate particularly on the Southern periphery all but rules out political debate both at the Union level and within each country about the principles that should guide fiscal policy. In this article, I discuss how filling that deficit depends on establishing some 'minimal sovereignty conditions' in the euro area, which is the only way to secure space for a 'safe' political discussion, free from asymmetric market pressures. Namely, eurozone members need to, at a minimum, formally commit the central bank to be the lender of last resort, and eliminate the financially destabilising features of the current banking union.

Orsola Costantini, United Nations Conference on Trade and Development, Geneva, Switzerland.


References

Arestis P McCauley K Sawyer M Commentary. An alternative stability pact for the European Union Cambridge Journal of Economics 2001 25 1 113 130 10.1093/cje/25.1.113
Bernanke, B. (2008), The Economic Outlook, Testimony before the Committee on the Budget, U.S. House of Representatives.
Biondi, Y. and C. del Barrio (2019), Unravelling economic solidarity: A systemic view on risk-sharing in the European banking sector, Financial Stability Conference-FSC Research Workshop.
Blanchard O Public debt and low interest rates, Presidential Address at the American Economic Association Annual meeting American Economic Review 2019 109 4 1197 1229 10.1257/aer.109.4.1197
Bordignon, M. (2020, 21 July), Recovery Fund, non è tutto oro quel che luccica, La Voce: https://www.lavoce.info/archives/68641/recovery-fund-non-e-tutto-oro-quello-che-luccica/ (17 September 2020).
Buchanan J. and R. Wagner (1977), Democracy in Deficit, The Political Legacy of Lord Keynes, in The Collected Works of James Buchanan, Volume 8, Liberty Fund.
Celi, G., A. Ginzburg, D. Guarascio and A. Simonazzi (2017), Crisis in the European Monetary Union: A core-periphery perspective, Routledge.
Cesaratto, S. and G. Zezza (2018), What went wrong with Italy, and what the country should now fight for in Europe, FMM Working Paper, 37.
Ciccone, R. (2013), Public debt and aggregate demand: some unconventional analytics, in Sraffa and the Reconstruction of Economic Theory, Volume 2, Palgrave Macmillan, 15–43.
Costantini, O. (2015), The cyclically adjusted budget: history and exegesis of a fateful estimate, Institute for New Economic Thinking Working Paper, 24.
Costantini O Political economy of the Stability and Growth Pact European journal of economics and economic policies: Intervention 2017 14 3 333 350
Costantini, O. (2018, 10 October), Why Hysteria Over the Italian Budget Is Wrong-Headed, Institute for New Economic Thinking blog, https://www.ineteconomics.org/perspectives/blog/why-hysteria-over-the-italian-budget-is-wrong-headed (17 September 2020).
Clancy, E. (2020), Discipline and Punish: End of the Road for the EU’s Stability and Growth Pact, A Report Commissioned by Martin Schirdewan MEP, https://emmaclancy.files.wordpress.com/2020/02/discipline-and-punish-eu-stability-and-growth-pact.pdf (17 September 2020).
Di Majo, A. (2020), Democrazia di bilancio e governo delle finanze pubbliche nella storia del budgeting pubblico, Società italiana di economia pubblica Working Paper, 752.
Dullien, S., C. Paetz, A. Watt and S. Watzka (2020), Proposals For A Reform Of The EU’s Fiscal Rules And Economic Governance, IMK Report, 159e.
Enria, A. (2019, February 18), Banking union — challenges ahead, Introductory remarks, session on Banking Union — Challenges Ahead, European Parliamentary Week.
European Fiscal Board (2019), Assessment of EU fiscal rules with a focus on the six- and two-pack legislation.
Ferguson, T. and E. Kane (2020, 16 May), Coronavirus Means Zero Hour for the European Union, Institute for New Economic Thinking blog, https://www.ineteconomics.org/perspectives/blog/coronavirus-means-zero-hour-for-the-european-union (16 September 2020).
Giacchè, V. (2017, 19 July), The Real Cause of the Italian Bank Bailouts and Euro Banking Troubles, Institute for New Economic Thinking blog, https://www.ineteconomics.org/perspectives/blog/the-real-cause-of-the-italian-bank-bailouts-and-euro-banking-troubles (16 September 2020).
Godley W Maastricht and all that London Review of Books 1992 14 19 2 3
Goodhart C A E The two concepts of money: implications for the analysis of optimal currency areas European Journal of Political Economy 1998 14 3 407 432 10.1016/S0176-2680(98)00015-9
Heimberger P Kapeller J The performativity of potential output: pro-cyclicality and path dependency in coordinating European fiscal policies Review of International Political Economy 2017 24 5 904 928 10.1080/09692290.2017.1363797
Heimberger P Potential Output, EU Fiscal Surveillance and the COVID-19 Shock Intereconomics 2020 55 3 167 174 10.1007/s10272-020-0895-z 32536715 PMC7276112
Horn, G. and A. Watt (2017), Wages and Nominal and Real Unit Labour Cost Differentials in EMU, European Commission DG ECFIN Discussion Papers, 2015-059.
International Monetary Fund (2018), Euro Area Policies. Financial Sector Assessment Program Technical Note — Bank Resolution and Crisis Management, IMF Country Report, 18/232.
Irvin, G. and A. Izurieta (2011), Fundamental Flaws in the European Project, Economic and Political Weekly, 14–16.
Lincei Committee on Covid-19 (2020, 9 May), The Covid crisis and a possible turning point for the European Union, Accademia dei Lincei.
Kane, E. J. (2017), Europe’s Zombie Megabanks and the Differential Regulatory Arrangements that Keep Them in Play, Institute for New Economic Thinking Working Paper Series, 64.
Kregel, J.A. (1985), Budget Deficits, Stabilisation Policy and Liquidity Preference: Keynes’s Post-War Policy Proposals, in F. Vicarelli (ed.), Keynes’s Relevance Today, 36–47, Macmillan.
Minenna M Dosi G Roventini A ECB monetary expansions and euro area TARGET2 imbalances: a balance-of-payment-based decomposition European Journal of Economics and Economic Policies: Intervention 2018 15 2 147 159
Myrdal, G. (1939), Fiscal Policy in the Business Cycle, American Economic Review, 29(1), Supplement, Papers and Proceedings of the Fifty-first Annual Meeting of the American Economic Association, 183–193.
O’Connell A European crisis: a new tale of center-periphery relations in the world of financial liberalization/globalization? International Journal of Political Economy 2015 44 3 174 195 10.1080/08911916.2015.1035986
Palumbo, A. (2013), Potential output and demand-led growth, in E. S. Levrero, A. Palumbo and A. Stirati (eds.), Sraffa and the Reconstruction of Economic Theory, 92–119, Palgrave Macmillan.
Parguez A The expected failure of the European economic and monetary union: a false money against the real economy Eastern Economic Journal 1999 25 1 63 76
Pasinetti L L The myth (or folly) of the 3% deficit/GDP Maastricht ‘parameter’ Cambridge Journal of Economics 1998 22 1 103 116 10.1093/oxfordjournals.cje.a013701
Prante F J Bramucci A Truger A Decades of Tight Fiscal Policy Have Left the Health Care System in Italy Ill-Prepared to Fight the COVID-19 Outbreak Intereconomics 2020 55 3 147 152 10.1007/s10272-020-0886-0 32536710 PMC7276098
Righi, S. (2020, 16 May), La sentenza di Karlsruhe tra diritto e politica, Pandora Rivista, https://www.pandorarivista.it/articoli/la-sentenzadi-karlsruhe-tra-diritto-e-politica/#_ftnref1 (24 September 2020).
Roncaglia, A. (2017, 8 February), The economist as an expert: a prince, a servant or a citizen?, Institute for New Economic Thinking blog, https://www.ineteconomics.org/perspectives/blog/the-economist-as-an-expert-a-prince-a-servant-or-a-citizen (17 September 2020).
Schnabel, I. (2020, 11 September), The shadow of fiscal dominance: Misconceptions, perceptions and perspectives” Speech at the Centre for European Reform and the Eurofi Financial Forum, https://www.ecb.europa.eu/press/key/date/2020/html/ecb.sp200911∼ea32bd8bb3.en.html (17 September 2020).
Seccareccia M Keynesianism And Public Investment: A Left-Keynesian Perspective On The Role Of Government Expenditures And Debt Studies in Political Economy 1995 46 43 78 10.1080/19187033.1995.11675366
Shapiro, N. (2017, 13 June), The Hidden Cost of Privatization, Institute for New Economic Thinking blog, https://www.ineteconomics.org/per-spectives/blog/the-business-of-government (16 September 2020).
Simonazzi, A. and A. Vianello (1999), Liberalizzazione finanziaria, moneta unica europea e occupazione, in F. R. Pizzuto (ed.), Globalizzazione, Istituzioni e Coesione Sociale, 227–253, Meridiana.
Storm S Naastepad C W M Europe’s Hunger Games: income distribution, cost competitiveness and crisis Cambridge Journal of Economics 2015 39 3 959 986 10.1093/cje/beu037
Storm S Lost in deflation: Why Italy’s woes are a warning to the whole Eurozone International Journal of Political Economy 2019 48 3 195 237 10.1080/08911916.2019.1655943
Tooze, A. (2020, August 7), It’s a New Europe — if You Can Keep It, Foreign Policy, https://foreignpolicy.com/2020/08/07/merkel-macron-eu-its-a-new-europe-if-you-can-keep-it/ (16 September 2020).
Truger A Austerity in the euro area: the sad state of economic policy in Germany and the EU European Journal of Economics and Economic Policies: Intervention 2013 10 2 158 174
Truger A Reviving fiscal policy in Europe: towards an implementation of the golden rule of public investment European Journal of Economics and Economic Policies: Intervention 2016 13 1 57 71
United Nations Conference on Trade and Development (2020), Trade and Development Report: From Global Pandemic to Prosperity for All: Avoiding Another Lost Decade.
Watt A Monetary financing of public investment: a viable way forward for the euro area? European Journal of Economics and Economic Policies: Intervention 2016 13 2 241 254
Weeks S Portugal in Ruins: From “Europe” to Crisis and Austerity Review of Radical Political Economics 2019 51 2 246 264 10.1177/0486613418776693
